# Redesigning Portable Health Clinic Platform as a Remote Healthcare System to Tackle COVID-19 Pandemic Situation in Unreached Communities

**DOI:** 10.3390/ijerph17134709

**Published:** 2020-06-30

**Authors:** Masuda Begum Sampa, Md. Rakibul Hoque, Rafiqul Islam, Mariko Nishikitani, Naoki Nakashima, Fumihiko Yokota, Kimiyo Kikuchi, Md Moshiur Rahman, Faiz Shah, Ashir Ahmed

**Affiliations:** 1 Department of Advanced Information Technology, Kyushu University, Fukuoka 819-0395, Japan; ashir@ait.kyushu-u.ac.jp; 2 School of Business, Emporia State University, Emporia, KS 66801, USA; mhoque@emporia.edu; 3 Medical Information Center, Kyushu University Hospital, Fukuoka 812-8582, Japan; rimaruf@med.kyushu-u.ac.jp (R.I.); makorin@info.med.kyushu-u.ac.jp (M.N.); nnaoki@med.kyushu-u.ac.jp (N.N.); 4 Institute of Decision Science for Sustainable Society, Kyushu University, Fukuoka 819-0395, Japan; yokota.fumihiko.785@m.kyushu-u.ac.jp; 5 Department of Health Sciences, Faculty of Medical Sciences, Kyushu University, Fukuoka 812-8582, Japan; kikuchia@hs.med.kyushu-u.ac.jp; 6 Graduate School of Biomedical and Health Sciences, Hiroshima University, Hiroshima 734-8553, Japan; moshiur@hiroshima-u.ac.jp; 7 Yunus Center, Asian Institute of Technology, Klong Luang, Pathumthani 1212, Thailand; fshah@ait.ac.th

**Keywords:** COVID-19, unreached communities, developing countries, portable health clinic, remote healthcare system

## Abstract

Medical staff carry an inordinate risk of infection from patients, and many doctors, nurses, and other healthcare workers are affected by COVID-19 worldwide. The unreached communities with noncommunicable diseases (NCDs) such as chronic cardiovascular, respiratory, endocrine, digestive, or renal diseases became more vulnerable during this pandemic situation. In both cases, Remote Healthcare Systems (RHS) may help minimize the risk of SARS-CoV-2 transmission. This study used the WHO guidelines and Design Science Research (DSR) framework to redesign the Portable Health Clinic (PHC), an RHS, for the containment of the spread of COVID-19 as well as proposed corona logic (C-Logic) for the main symptoms of COVID-19. Using the distributed service platform of PHC, a trained healthcare worker with appropriate testing kits can screen high-risk individuals and can help optimize triage to medical services. PHC with its new triage algorithm (C-Logic) classifies the patients according to whether the patient needs to move to a clinic for a PCR test. Through modified PHC service, we can help people to boost their knowledge, attitude (feelings/beliefs), and self-efficacy to execute preventing measures. Our initial examination of the suitability of the PHC and its associated technologies as a key contributor to public health responses is designed to “flatten the curve”, particularly among unreached high-risk NCD populations in developing countries. Theoretically, this study contributes to design science research by introducing a modified healthcare providing model.

## 1. Introduction

Beginning at the end of 2019, the COVID-19 outbreak was declared a pandemic by WHO on 11 March 2020 [1,2]. The main symptoms of COVID-19 are fever, cough, sore throat, and respiratory complications [3]. Respiratory infections can be transmitted through droplets of different sizes, and according to current evidence, the COVID-19 virus (SARS-CoV-2) is primarily transmitted between people through respiratory droplets (with a particle size of >5–10 μm) when a person comes in close contact (within 1 m) with someone who has respiratory symptoms [4,5]. Moreover, other contact routes, such as the immediate environment around the infected person, may also cause transmission of the virus [6]. In the case of cluster pneumonia of unknown etiology, health workers are recommended droplet and contact precautions when caring for patients and airborne precautions for aerosol-generating procedures conducted by health workers [7].

SARS-CoV-2 became a pandemic virus due to a multitude of factors such as the early spread of the virus by asymptomatic carriers, uncontrolled social behaviors, and insufficient personal protective equipment (PPE), and both the advanced and developing medical systems appear overwhelmed. Public health experts are working at relieving pressure on healthcare facilities so resources can be focused on COVID-19 patients. The crisis is even more threatening for developing countries with large underserved populations. As cases multiply, governments, irrespective of developed and developing countries, restricted “nonessential” services by declaring a state of emergency not due to the fear of contagion but as a procedural protocol in order to contain the virus by mandating social distancing. 

The Bangladesh government imposed a nationwide lockdown since 26 March 2020 to curb the spread of the novel coronavirus [8]. Bangladesh with its 165 million inhabitants and a density of 1265 people per sq. km is in a great crisis as the outbreak could spread further. In Bangladesh, 70% of people live in rural areas where medical facilities are almost absent. Recently, the most serious problems in Bangladesh are the lack of personal protective equipment (PPE) for doctors or its low quality as well as too many people staying in close proximity and sick people hiding their symptoms. The situation has become more vulnerable as there are an estimated 5.2 healthcare workers (doctors and nurses) available per 10,000 people [9]. As of 13 June 2020, at least 78,052 people have been infected [10] among which more than 4% are the healthcare workers, whereas 2.5% healthcare workers are affected worldwide [11]. Most private medical facilities in Bangladesh are turning away patients with other health issues amid the coronavirus outbreak even though the government has issued a circular threatening of the annulment of their license to operate [12,13]. This has deprived many non-COVID-19 patients of needed treatment, resulting in death from cough, fever, breathing difficulties, and diarrhoea [14].

Older people and people with noncommunicable diseases (NCDs) such as cardiovascular disease, diabetes, chronic respiratory disease, and cancer appear to be more vulnerable to becoming severely ill with COVID-19. The COVID-19 mortality rate in China with the presence of one or more preexisting NCD conditions is represented in Table 1. It shows that almost every fatality is associated with preexisting NCDs, while no preexisting medical conditions had a fatality rate of only 0.9%. Therefore, preexisting NCD-related illnesses increase the risk of death. According to data from Italy, almost 99% of deaths from COVID-19 were related to preexisting diseases, and of these, hypertension accounted for 75%. As such, the public health impact of COVID-19 will include a significant number of NCD-associated mortalities. 

The control strategy of large-scale and prolonged lockdowns is bound to increase the morbidity risk for those living with NCDs even more. Disrupting regular exercise and monitoring check-ups and adding to mental stress may further undermine immune systems among such patients and impact morbidity and mortality rates. Many researchers are focusing on the effect of COVID-19 on mental health [16], while there is growing public awareness of the association of NCDs with COVID-19 mortality rates, and hence, there is also the need to highlight the negative impact of uncontrolled NCDs among populations over the long-term. 

Remote Healthcare Services (RHS) appear well-suited to this purpose, providing NCD patients in remote locations access to critical monitoring services without increasing risk of infection by visiting a hospital. Portable Health Clinic (PHC) services, which is an RHS, have proven efficacy in providing necessary information and preventive measures for people without access to healthcare facilities [17,18,19]. PHC systems have been developed in a preventive healthcare approach with a special focus on noncommunicable diseases. It appears necessary to modify the strategy-of-use of the PHC to better respond to the healthcare management needs of NCD patients in an emergency created by the pandemic, particularly in developing countries. In a lockdown situation, this platform can be effectively used to control and manage patient triage, thus relieving pressure on hospitals and healthcare facilities. 

Challenges of remote healthcare systems during an emergency like disasters, pandemics, etc. implies unique challenges to healthcare delivery [20]. In the context of developing countries, the scenario of using eHealth technology is completely different, especially for rural people who are typically low health-literate and are more at risk for NCDs. Nearly every one of the 205 countries affected by COVID-19 has instituted social distancing measures. Many, including Bangladesh, China, France, India, Italy, Korea, Pakistan, Singapore, Spain, Taiwan, and Thailand, have enforced large-scale lockdowns to avoid spikes in cases and to buy time to set up appropriate responses. During such emergencies, RHS platforms assume even greater relevance, especially for NCD patients who may be turned away from hospitals treating acute patients. The real challenge is providing primary care services to NCD patients within the context of social distancing. Leveraging the PHC and attendants of eHealth technologies already successfully deployed in support of rural and remote patients makes this challenge surmountable [21].

Some previous research already confirms the effectiveness of eHealth in emergency response situations, primarily for urban areas in developed countries. However, healthcare planners agree on the need to monitor NCD patients in rural populations of developing countries in the pandemic situation [22]. However, some questions remain unanswered:

- How to redesign RHS such as the PHC platform to achieve the goal more effectively in a pandemic situation like COVID-19?
- How to ensure coverage of underserved rural populations who have comparatively less access to healthcare facilities?
- How can the RHS platform like PHC be adapted to accommodate emergency response situations like COVID-19?

Therefore, this paper tries to answer the above research questions and presents the process of designing and developing an RHS based on the general requirements to tackle communicable diseases for allowing both COVID-19 patients and non-patients in Bangladesh. No previous study to date has examined the scopes of designing and developing an RHS based on the general requirements to facilitate primary screening and triaging COVID-19 and primary healthcare services for preventing COVID-19 and controlling NCDs. However, such screening and triaging COVID-19 by an RHS is important for cost-effective check-ups and for reducing the risk of transmission for unreached communities with various needs.

## 2. Evaluation of eHealth System During Emergency

Extensive research works are conducted only on the hospital information systems to construct the hospital management information system of infectious diseases. To improve the efficiency and level of infectious disease management of the hospital, those research investigate their risk factors, the rules of emergence, and the control measures for infectious disease management [23,24,25]. However, a challenge during the pandemic in progress is to identify the determinants underpinning the spatial and temporal patterns of the epidemic for making preventive strategies by the decision-makers [26]. Along with these, health services for reducing transmission and triaging is also a necessity. 

The provision of effective eHealth services likely enhances patients’ own abilities to manage their NCDs during the COVID-19 outbreak, especially in places where lack of sanitation or availability of PPE increases the risk of contagion. More importantly, eHealth solutions minimize direct contact between the public and healthcare providers and thus promote social distancing without affecting the strength of patient support [27]. Consultancy over video communications has become useful for the delivery of preventive and consultation services. Remote consultancy over phone or video communication has already shown social, technical, and commercial benefits for the management of NCDs.

The important benefits of telemedicine for the health systems for handling COVID-19, especially on monitoring, surveillance, and detection and the potential for machine learning and artificial intelligence, have been focused very well in many articles starting from one of the very first ones but an opinion from the patients’ side is not reported well yet, though it is of importance to draw attention to the ongoing importance of patient involvement when it comes to urgent eHealth solutions for COVID-19 [20,28,29]. During public health emergency like COVID-19 pandemic, the digital infrastructures remain intact and doctors can still be in touch with patients but yet no large-scale telemedicine services for monitoring acute and chronic patients’ health status and for allowing continuity of care have been considered in the highly affected countries like Italy [30].

With the importance of eHealth becoming formally recognized, several governments are reinterpreting regulations to enable remote medical services by licensed practitioners. The governments are supplementing healthcare budgets to counter the impact of the pandemic, such as the Medicare Benefits Schedule [31] and Medicare in the United States, expanding the coverage range for the testing and treatment of COVID-19 without subscribers’ expense [32]. This allocation can support a range of eHealth services during the COVID-19 phase, enabling more people to receive healthcare at a significantly lower cost compared to hospital-centric services, including telehealth consultations with general practitioners and specialists. Doctors or nurses manning the eHealth service will be able to guide patients over video communication.

Healthcare systems have had to adapt rapidly to the evolving situation for three main reasons: firstly, there is a need to triage and treat large numbers of patients with respiratory illness [27]; secondly, there is a need to protect the healthcare workforce to ensure they can treat the sick [33,34]; and thirdly, we need to shield the elderly and most vulnerable from becoming infected [35].

## 3. Design Methodology

This study used the WHO guidelines to tackle COVID-19 as a theoretical basis of the designed service to satisfy the general requirements in the service and also followed the Information System Research (ISR) framework to involve the people in the service design and evaluation phase. The guidelines of WHO explains the key components of required healthcare services for COVID-19 disease. According to WHO guidelines [36,37,38], the following are the key components of required healthcare services for COVID-19 disease:

### 3.1. Primary Screening and Triage

WHO recommends screening and isolation of all patients with suspected COVID-19 at the first of point of contact with the health care system, such as outpatients and emergency departments/clinics. Early detection of suspected patients allows for the timely initiation of appropriate prevention and control measures [36,37].

### 3.2. Prevention and Control (Isolation and Quarantine)

Isolation is a long-established containment response that is designed to prevent further transmission from an individual suspected of exposure to a contagious disease. Suspected infectious individuals not in immediate need of medical attention may be effectively quarantined at home instead of a hospital. In pandemics, it is often impossible to accurately identify cases and carriers of the disease, and hence, the closure of premises such as schools, markets, theatres, etc. are declared to physically limit further transmission.

### 3.3. Traceability and Privacy

Physical contact, direct or indirect, is the most important channel for the transmission of infectious disease. Contact tracing involves identifying everyone who may have had exposure to an initial case and tracing it to all possible contacts. The privacy of the patients’ needs to be maintained to avoid any sort of discrimination to the patient or his/her family.

## 4. Results

### 4.1. Redesigned PHC for COVID-19 

To design a useful information system-based healthcare service based on the WHO guidelines, we resorted to following the directions and guidelines as proposed in Information System Research (ISR) framework [39]. 

Theorizing in design science research (DSR) is different than other types of science. It has two general modes of DSR activity and theorizing: (i) the interior mode, where theorizing is done to formulate a theory for design and action with the prescriptive statement about the way to design the artifact, and (ii) the exterior mode, where analyzing, describing, and predicting are done on what happens to the artifacts in the external environment [39,40]. We designed our PHC following the theories of the DSR framework. In PHC architecture, all artifacts or medical devices are organized following the prescriptive roles provided by WHO. 

**Figure 1:**
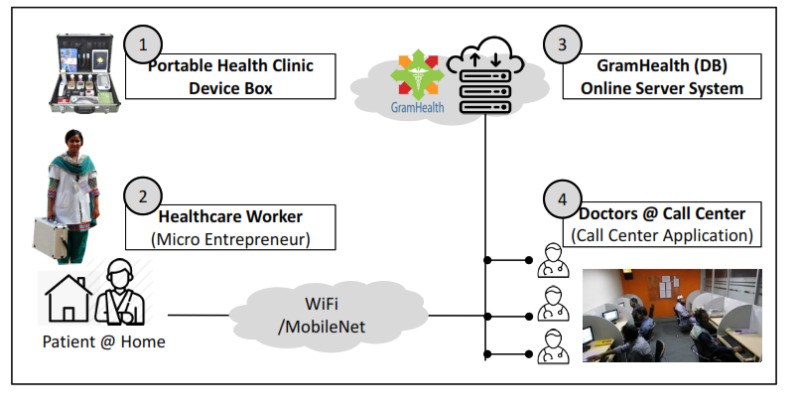
Portable Health Clinic (PHC) system architecture.

A Portable Health Clinic (PHC) system (shown in Figure 1) has been developed as an RHS system for the unreached communities with a special focus on noncommunicable diseases [41,42]. A health worker visits a patient with the PHC box to measure vital information and to upload the data with the medical history of the patient to an online server by using the GramHealth Client Application. The remote doctor gets access to this data and makes a video call to the patient for further verification. Finally, the doctor creates an online prescription and preserves it on the online server under the patient’s profile. The health worker accesses the system, prints the prescription from the server, and passes it to the patient with detailed explanation instantly. The whole process takes about 15 to 30 minutes per patient. The PHC system introduces a triage system to classify the subjects in four categories, namely, (i) green or healthy, (ii) yellow or suspicious, (iii) orange or affected, and (iv) red or emergent, based on the gradual higher risk status of health. The subjects under orange and red who are primarily diagnosed as in the high-risk zone need a doctor’s consultation.

PHC was initially designed to provide primary health screening services to the unreached community in remote areas. It is time to test its compatibility in emergencies to lessen the mortality and morbidity due to NCDs in developing countries. The prevalence of NCDs such as diabetes, blood pressure, and chronic diseases may rise due to mental stress, fear, income loss, physical inactivity, and more food consumption and during the lock-down situation at home. In the spread of COVID-19, people can neither go out for physical exercises such as morning or evening walking nor visit a hospital for NCDs during the lockdown situation.

**Figure 2:**
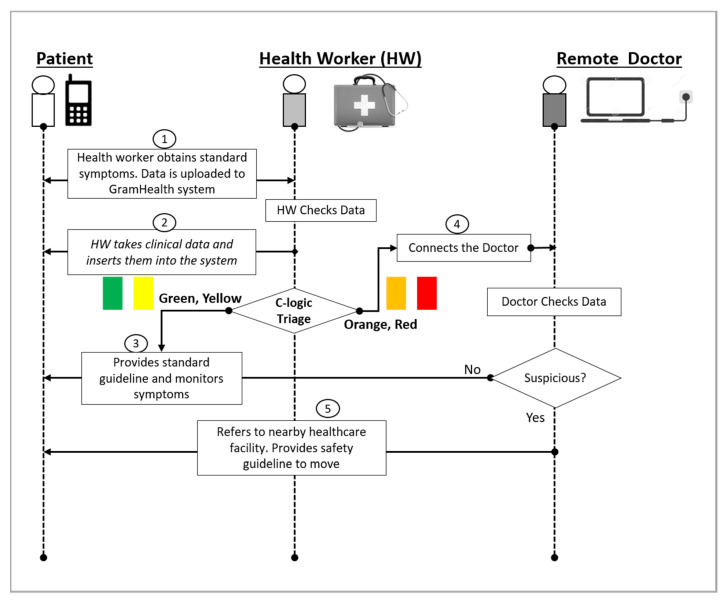
Primary screening and triaging COVID-19 potential patients by using Portable Health Clinic (PHC).

This PHC system is modified to be used for addressing communicable diseases like COVID-19. The steps are explained in Figure 2. At first, a potential patient can place a call to the nearby health worker. The health worker can ask questions as per the standard protocol. A patient with a smartphone can fill in a self-check form through the web, which ultimately goes to the nearby community health worker. The health worker checks the data and visits the patient with the PHC box for clinical measurements. Although the original PHC box contains various medical sensors, only COVID-19-related sensors will be used. These are (i) thermometers (OMRON) for measuring body temperature; (ii) pulse oximeters (OXI METER) for measuring oxygenation of blood (SpO_2_); (iii) digital blood pressure (BP) machines (A&D) for measuring blood pressure, pulse rate, and arrhythmia; and (iv) glucometers (TERUMO) for measuring blood glucose in the case of diabetic patients. After taking the measurements, the triage algorithm at the PHC client device will run to classify the patient into four categories. Table 2 shows the proposed logic set for COVID-19. The orange and red marked patients will be connected with the remote doctor. The doctor will have a video conversation with the patient for further verification of their status. 

### 4.2. Primary Screening and Triaging: Questionnaire and Measurement

Unlike the conventional PHC for NCDs, the local health worker collects the primary symptoms of a patient through a standard questionnaire. If the patient is identified as a potential patient, the PHC COVID-19 box will be sent to the patient’s home temporarily together with an operation manual so that they can check themselves under the guidance of the health worker. This will save both parties from infection. 

Now, if a patient is identified as a potential COVID-19 carrier by this primary screening using the triage system, as shown in Table 2, the patient will be immediately advised to see the nearby hospital for further investigation and follow-up as needed. Otherwise, the health worker will provide a guideline to stay safe at home. Since the community health workers are already known to the patients, patients feel more comfortable and safer under their guidance. The privacy of sensitive information of patients will be protected and secured because it is required by an increasing body of legislative provisions and standards [43].

Table 3 shows the functionalities of a portable health clinic to meet general requirements.

### 4.3. Isolation and Quarantine

The high-risk potential patients are dealt with by the hospital, and they can go under treatment or isolation in hospital or home quarantine as per the result of the Polymerase Chain Reaction (PCR) test. On the other hand, the health worker can also guide the remaining patients if they need home quarantine based on the primary screening. Thus, the spread of the highly contaminating COVID-19 can be efficiently controlled with the utilization of local health workers.

### 4.4. Traceability and Privacy

In PHC service policy, the health workers are usually from the respective localities. Therefore, they know their communities and are in a position to trace with ease and speed those exposed to direct or indirect contact. Since the PHC reaches people at their doorstep, only those referred by the doctor online need to go to the hospital. This process helps maintain privacy as well.

## 5. Discussion

In its existing functional form, deploying the PHC and related RHS technologies for socially distanced populations during a public health emergency, such as the COVID-19 pandemic, is beneficial in reducing the risk of transmission to frontline healthcare professionals. Moreover, findings indicate that frontline medical staff experience heightened levels of stress when coming into direct contact with COVID-19 patients. The impact of stress on cardiovascular function is well-established experimentally, independent of known risk factors associated with NCDs [44,45,46,47,48]. The PHC service may create an effective physical separation between the caregiver and the patient without materially diminishing the quality of care or the reliability of care management responses. 

In Bangladesh, medical staffs such as doctors, nurses, and volunteers who are fighting the coronavirus are being socially excluded, driven from the flats or rooms they rent and banned from his or her buildings. Official reports from China indicate that 71.5% of the frontline healthcare providers treating COVID-19 patients experience high levels of mental stress. Also, 50.4% show signs of depression, 44.6% exhibit anxiety states, and 34.0% suffer from insomnia [49]. Fatalities among healthcare professionals reported from China, Korea, Pakistan, and the United Kingdom may well be causally linked to reduced efficiency as a result of anxiety-induced stress as well as lack of sleep and depressive states [50]. In Japan too, healthcare staff treating the new COVID-19 patients report higher mental stress compared to routine care assignments [51]. PHC and attendant RHS technologies can create the required physical distancing that increases the sense of safety among medical staff and is likely to reduce stress. 

The most conclusive method for determining COVID-19 infection utilizes PCR techniques, which require running the patients’ DNA sample through specialized equipment in a laboratory environment. At present, therefore, the remote diagnosis of COVID-19 is not possible. However, the PHC can reliably triage individuals presenting with symptoms associated with COVID-19 using a checklist released by the Centers for Disease Control (CDC) [52]. This way, the PHC can help stem the unbridled flow of concerned citizens to healthcare facilities. This reduces the burden on already overextended healthcare staffs and facilities but still allows the concerned citizen to receive reliable well-being information from the PHC worker and retains human contact essential to medical care [53]. While conventional telemedicine applications only offer live contact with a medical professional, the PHC system incorporates diagnostic testing for screening NCDs and nutritional status. A unique aspect of the PHC system is its built-in algorithm that compares up to 17 diagnostic parameters in real-time and generates a triage plan that is relayed to the doctor manning the telemedicine call-point. This eliminates any interference by the PHC worker attending the patient by providing the attending doctor with direct control over patient management decisions. PHC can reduce the risk of transmission to frontline healthcare workers, can reduce psychosocial stress on frontline healthcare staff, and can optimize healthcare resources for more patients who need them most.

As part of its COVID-19 response, the United States Congress has promulgated Public Law No: 116–123, which provides for the temporary removal of restrictions on telehealth services for Medicare beneficiaries [54]. These developments indicate that the PHC system can be adapted to regulatory and best practice parameters, either by securing the clinical role of the licensed medical practitioner within the delivery model or by modulating the level of service provision in ways that do not impinge on best practice guidelines.

In summary, the PHC, even in its present form, can be effectively deployed to eliminate the risk of transmission among frontline healthcare staff and to contribute significantly towards reducing pressure on healthcare services and resources. With considered realignment of its technical configuration, the PHC can be deployed as an ancillary resource supporting large-scale public health emergencies, exemplified by the COVID-19 pandemic.

The PHC system can be effective in providing the following:

(1) a primary-level screening mechanism that can demonstrably reduce the burden of NCD-related complications among COVID-19 patients and that can directly contribute to the reduction of the incidence of NCDs by timely advice and treatment;
(2) a primary healthcare service platform for underserved populations in remote regions of developing countries and now mature enough to be adapted to respond to large-scale public health emergencies such as COVID-19 to impact the reduction of associated mortality and morbidities;
(3) a reliable platform for early detection of NCDs and associated comorbidities among target populations and for effectively contributing to a tangible reduction in the burden of disease;
(4) a key ancillary mechanism for controlling patient-to-caregiver transmission of COVID-19 by creating physical distance between all except diagnosed cases and attending clinical staff;
(5) evidence for health authorities to choose eHealth technologies, such as a PHC service, to provide primary healthcare services simultaneously for COVID-19 and NCDs, including video consultation with physicians, preventive health education, and awareness at the grassroots, and to encourage well-being behaviors;
(6) an effective outreach tool for controlling NCDs and for decreasing the burden of disease on the target community;
(7) a new approach to responding to large-scale public health emergencies like COVID-19 and to contributing directly to building adaptive resilience among populations at risk.

## 6. Limitations

If the paraprofessional worker visiting homes is not well-trained in self-disinfection or access to disinfection facilities is not available between one visit and another, then the contagion can be transmitted by the paraprofessionals. This is indeed what has happened in nursing homes and assisted living facilities across the United States and Japan. This may potentially facilitate the spread of the virus rather than containment. 

However, the main challenge for deploying the PHC during large-scale public health emergencies such as COVID-19 is ensuring that the patient is amenable to self-checking, guided by the PHC health worker. Initial screening requires 3 simple tests for which a manual is provided. Health workers can also guide online. Another challenge is to ensure access to a facility equipped for a definitive diagnostic test, such as the PCR test in the case of COVID-19 so that the diagnosed patient can be triaged to a hospital for treatment.

## 7. Conclusions

This paper touched upon relevant current and future public health implications arising from the COVID-19 outbreak. It provided an overview of how several centralized initiatives have emerged to tackle the situation. Our initial examination of the suitability of the PHC and its associated technologies as a key contributor to public health responses designed to “flatten the curve”, particularly among unreached high-risk NCD populations in developing countries, indicates the strong possibility of affirmative impact. 

In this paper, we redesigned the existing PHC for the containment of the spread of COVID-19 as well as proposed corona logic (C-Logic) for the main symptoms of COVID-19, such as fever, cough, sore throat, respiratory complications, etc. Through modified PHC service, we can help people to boost their knowledge, attitude (feelings/beliefs), and self-efficacy to execute preventative measures. Knowledge about COVID-19 means what are the causes, sources of infection, symptoms, ways of transmission, and prevention. As it is a new disease and has become a pandemic within a short period, there is a lack of knowledge, especially among rural people. Therefore, it is very important to fill these knowledge gaps timely to prevent and control the spread, which will lead to better practice for prevention and control of the contagious disease. 

Portable health clinics introduce an affordable, usable set of sensors with the transmission facility to convey clinical data to the remote doctor so that the doctor can make an accurate decision. PHC with its new triage algorithm (corona logic) classifies the patients on whether the patient needs to move to a clinic for a PCR test. As mentioned in the previous sections, the new model can reduce the risk of transmission and psychological stress on frontline healthcare staff and can optimize healthcare resources for more patients who need them the most. The consultancy service is mostly on introducing nearby hospitals, providing doctor appointments, and interpreting prescriptions.

The salient point is that the same model can work in other countries both rural or urban to bring similar benefits for an emergency to reduce the transmission of diseases. Therefore, governments and other healthcare sectors can take initiative to use RHSs such as the PHC service, to provide primary healthcare services simultaneously for triaging susceptible COVID-19 and for supporting NCD patients isolated in various geographical locations.

Theoretically, this study contributes to design science research by introducing a modified healthcare providing model with a new triage logic.

## Figures and Tables

**Table 1:** Death rates with preexisting medical conditions in China [1,15].

Preexisting Conditions	Confirmed Death Rates	All Cases (Suspected and Asymptomatic) of COVID-19
Cardiovascular disease	13.2%	10.5%
Diabetes	9.2%	7.3%
Chronic respiratory disease	8.0%	6.3%
Hypertension	8.4%	6.0%
Cancer	7.6%	5.6%
No preexisting conditions		0.9%

**Table 2:** C-Logic: corona logic designed for a portable health clinic system.

No.	Symptoms	Healthy	Suspicious	Affected	Emergent
		Green	Yellow	Orange (Consultation)	Red (Emergency)
1	Fever	<37.5 °C	≥37.5 °C, Continue ≤3 days *	≥37.5 °C, Continue ≥4 days **	
2	muscle or joint pain	No	Continue ≤3 days *	Continue ≥4 days **	
3	Sore throat	No	Continue ≤3 days *	Continue ≥4 days **	
4	Dyspnea	No	Light to Moderate, Continue ≤3 days *	Light to Moderate, Continue ≥4 days **	
				or Severe	
5	Shortness of Breath	≤15/min	≤20/min	≤25/min	≥26/min
6	Cough	No	Continue ≤3 days *	Continue ≥4 days **	
7	Chillness	No	Continue ≤3 days *	Continue ≥4 days **	
8	SpO_2_ (%)	≥96%	≤95%, With no Symptom	≤95%, With Light to Moderate Dyspnea	≤95%, With severe Dyspnea
9	Fatigue	No	Light to Moderate, Continue ≤3 days *	Light to Moderate, Continue ≥4 days **	
				or Severe	
10	Loss of appetite	No	Continue ≤3 days *	Continue ≥4 days **	
11	Diarrhea	No	Continue ≤3 days*	Continue ≥4 days **	
12	Loss of taste	No		Yes	
13	Loss of smell sense	No		Yes	

Note: “3 days *” should be replaced by “1 day” and “4 days **” should be replaced by “2 days” for 65 years old or older patients with NCDs such as diabetes, heart failure, COPD, etc. who are using hemodialysis, immunosuppressants, and anticancer agents. No. 8, SpO_2_ is optional.

**Table 3:** Portable health clinic functionalities during general mode and emergency mode.

Activity	General Mode	Emergency Mode
Symptom Collection	Health Worker	Mobile Phone or App
Clinical Measurements	Health Worker	Patients Self-Test or Health Worker
Medical Consultancy	Remote Doctor	Remote Doctor
ePrescription	GramHealth Application, printed by eHealth worker	GramHealth Application or Medical Facilities
